# Metabioethics and ChatGPT-based posthumous memories at the service of palliative medicine

**DOI:** 10.31744/einstein_journal/2025CE1590

**Published:** 2025-07-02

**Authors:** Antonio Fábio Medrado de Araújo

**Affiliations:** 1 Center for Transdisciplinary Research in Bioethics Faculdade de Medicina da Bahia Universidade Federal da Bahia Salvador BA Brazil Center for Transdisciplinary Research in Bioethics, Faculdade de Medicina da Bahia, Universidade Federal da Bahia, Salvador, BA, Brazil.

Dear Editor,

Biocomputer literature has speculated on ChatGPT application in *post mortem* interactions, *i.e*., the interlocution between living people and supervening “memories” of already deceased persons.^1,2^ Within the generative artificial intelligence (AI) research spectrum, such speculations raise a bioethical dilemma related to the violation of the dignity of dead people, who did not consent or authorize such invasive appropriation and exploitation of their memories and *ethos* in their lives. In a sense, our perspective presupposes and proposes a new question: is the bioethical justification of the referred ChatGPT usefulness possible? Behold an approach with its relevance relying on metaphysical insight that individual autonomy contains a *post mortem* projection: perhaps this is a core statement of metabioethics (or bioethical metaphysics).

Any approach seeking for bioethical justification for ChatGPT technology application in *post mortem* interactions potentially significantly impacts palliative medicine. Indeed, it can lead terminally ill patients to improve their own autonomy by encouraging them to customize, train or personalize ChatGPT so that such a tool could enable the *post mortem* interlocution of their memories (entropic audiovisual records on key issues) with friends and relatives. Conjecture that might trigger positive effects regarding end-of-life perception/consciousness as a relevant existential value.^3^ Here is a clear unfolding of the essay “Castling Against Death: A Chess-Based Insight into the Paradox of Physician-Assisted Suicide”.^4^

**Figure 1:**
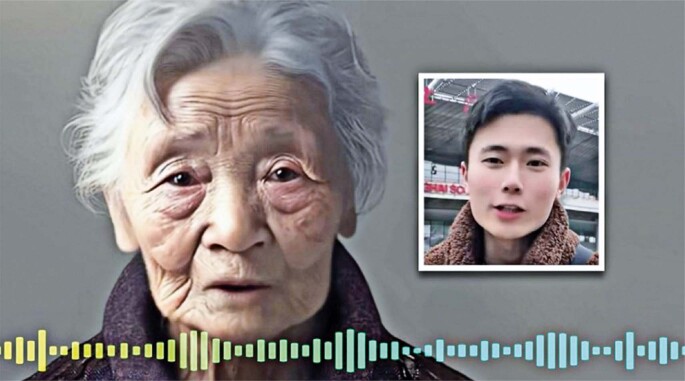
In China, a young man uses AI/chatbot to develop a “Matrix” audiovisual interaction with the avatar of his late grandmother (AI-generated persona)

Source: Yan A. Dead chat: Shanghai man uses AI technology to “resurrect” late grandmother by creating virtual version to talk to, triggering controversy in China. Sout Chin Mor Post, Shanghai, 17 April 2023 [cited 2025 Apr 24]. Available from: https://www.scmp.com/news/people-culture/trending-china/article/3216945/dead-chat-shanghai-man-uses-ai-technology-resurrect-late-grandmother-creating-virtual-version-talk^(2)^

**Figure 2:**
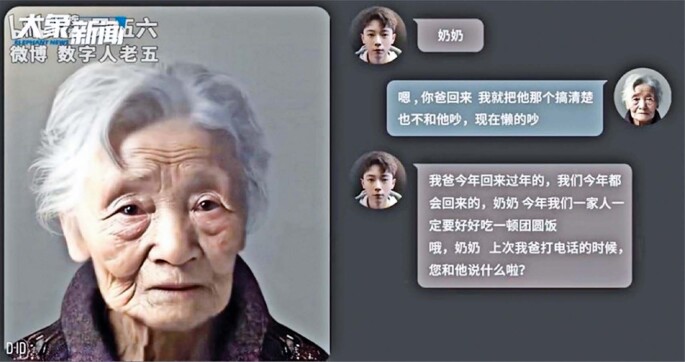
The same young man uses AI/chatbot to perform a textual dialog with the avatar of his late grandmother

Source: Yan A. Dead chat: Shanghai man uses AI technology to “resurrect” late grandmother by creating virtual version to talk to, triggering controversy in China. Sout Chin Mor Post, Shanghai, 17 April 2023 [cited 2025 Apr 24]. Available from: https://www.scmp.com/news/people-culture/trending-china/article/3216945/dead-chat-shanghai-man-uses-ai-technology-resurrect-late-grandmother-creating-virtual-version-talk^(2)^

**Figure 3:**
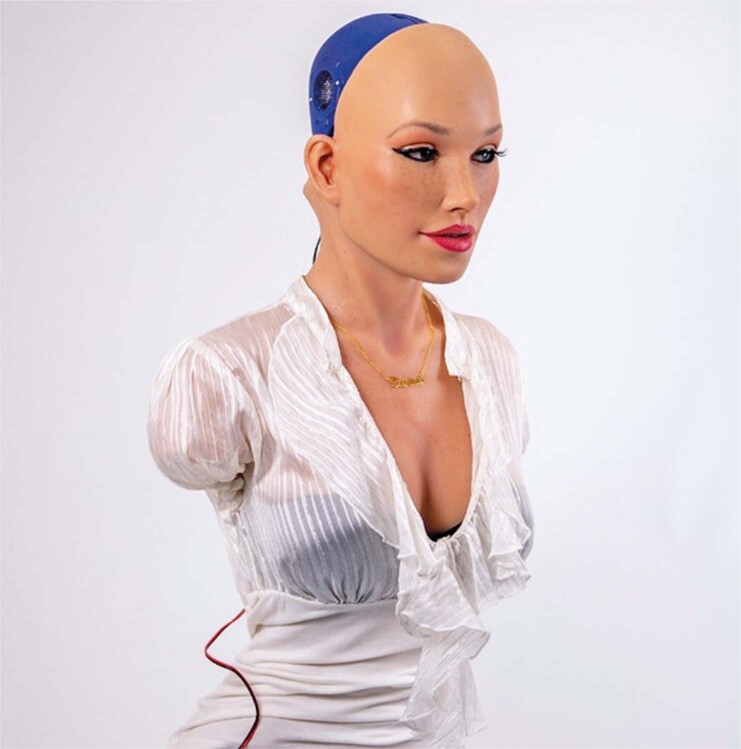
Sex android Denise

Source: Araújo A. Anthropomorphic sex robots across the genitalia-computer interface: AI-generated lover persona, infopower feminist bioethics, and Alexa-style humanity. AI Ethics. 2025;5:3383-6.^(8)^

Nonetheless, how to perform that in terminally ill patients retaining limited autonomy due to neurological impairments (*e.g.*, dementia or Asperger’s syndrome, the latter of being a form of an autism^5^ spectrum disorder)? We might deduce at least one hypothetical implication (emerging brain-reading medical technology-linked AI for palliative purposes) related to memory back-up, widening and re-signifying the intervention horizon of palliative care physicians. Brain-computer interface would be modelled on a device^6,7^ (*e.g*., electrode grids inside the cortex) that uses algorithms for tracking, scanning, translates and converts neuronal data into pictogram/pixel sets (non-verbal^5^ ludic communication) adapted to architecting display memories through a custom-made ChatGPT, associable to an immersive virtual space (metaverse) with the holographic avatar of a terminally ill patient (Figures 1 and 2) or interactive cyberspace (multiverse) via a phenotypically similar android, exhibiting similar morphology to that of anthropomorphic sex robot^8^ prototypes (Figure 3).

Brain-computer interfaces are already often implanted as assisted living devices for individuals with behavioral, language, sensorimotor, or cognitive disabilities, within neuroprosthetics, a multidisciplinary field at the interface of neurosciences and biomedical engineering, aiming at replacing parts of the nervous system compromised by neurological disorders or injury.^9^ Recent proof-of-concept studies suggest that electrical neuromodulation strategies could also be useful in alleviating/palliating certain cognitive and memory deficits, particularly concerning dementia.^6,9^

Related to the above-mentioned “dead chat” experiment (Figures 1 and 2), the adherence of terminally ill patients to design and calibrate a personalized chatbot reveals several important comparative advantages:

- higher-level bioethical legitimacy: due to prior consent and the most possible extent of autonomous participation of terminally ill patients;
- increased authorial credibility: due to the increased verisimilitude of *post mortem* feedback (resulting from algorithmic consciousness trained and sanctioned by the terminally ill);
- improved regulatory transparency: due likely to the scrutiny of medical associations and research ethics committees or a forthcoming National Palliative Care Authority (health policy related to interface of medtech and society), especially when e.g., the planning of chatbots precedes physician-assisted suicide;
- wider palliative range: due to the reliability/versatility acquired in palliating post-traumatic grief, mainly among the relatives of terminally ill patients.

Perpetuating the simulated memory (with higher accuracy or authenticity over time by optimizing data curation, toxicity filters, etc.)^10-12^ as the family legacy of terminally ill patients could become a smart and organic property/challenge of the semantic corpus of chatbots, like self-taught and continuous flow synapses. In addition, it frames a long-term journey to create a care-driven digital ancestry ecosystem (AI-generative “grandparents” would still manage to scour the web for everything from clinical trial outcomes to fake news/deepfakes involving their living descendants). All with the prior and direct consent of terminally ill patients, or indirect^13^ authorizations via legal representatives.
